# Practice Patterns of Palliative Radiotherapy for Advanced Cancer at a Large Institute in Saudi Arabia

**DOI:** 10.1089/pmr.2025.0008

**Published:** 2025-04-29

**Authors:** Wsam Ghandourh, Zaheeda Mulla, Belal Sharaf, Elham Ghabashi, Anan Bamakhrama

**Affiliations:** ^1^ Department of Health Care Management, Umm Al-Qura University, Mecca, Saudi Arabia.; ^2^ Department of Oncology, King Faisal Specialist Hospital & Research Centre (KFSH&RC), Jeddah, Saudi Arabia.; ^3^ Department of Palliative Care, Jeddah First Health Cluster, East Jeddah Hospital, Jeddah, Saudi Arabia.

**Keywords:** advanced cancer, palliative radiotherapy, practice patterns, utilization

## Abstract

**Background and Aims:**

Palliative radiotherapy practice patterns have been reported to vary widely, with a notable underutilization of single fraction treatment schedules. This study aims to investigate the outcomes and care patterns among patients receiving palliative radiotherapy for advanced cancer at a high-volume institution in Saudi Arabia.

**Materials and Methods:**

Electronic records were used to identify patients receiving palliative radiotherapy for advanced cancer between 2018 and 2023. Univariate analyses were used to assess tumor and patient factors potentially associated with single fraction use, including primary tumor, target site, sex, age, admission status, and geographical remoteness from the center. Survival outcomes were analyzed using Kaplan–Meier curves.

**Results:**

A total of 792 patients receiving 990 radiotherapy courses were identified. 60% of patients were female and 40% were male. The median age was 56.5 years (16.4 standard deviation [SD]). The most common primary histology was breast (34%), followed by gastrointestinal (13%). Single fraction treatment schedule represented 28.7% of all treatments and were most commonly used for extremities (*p* < 0.05). Multiple-fraction treatment schedule was more likely to be used for breast, chest, head-and-neck, pelvis, and spine *(p* < 0.05). The median survival was 6.9 months (SD = 8.9 months) and 25% of patients died within 30 days following radiotherapy. Median survival was shorter for male gender, admitted patients and those who did not complete their course of treatment (log-rank *p* < 0.05).

**Conclusion:**

Single fraction radiotherapy is underutilized in the management of advanced cancer patients, particularly those with bone metastases. Further research is warranted to develop clinical decision-making tools that enhance adherence to clinical guidelines and optimize treatment outcomes.

## Key Message

Despite guideline recommendations, single fraction radiotherapy remains underutilized for advanced cancer patients, particularly those with bone metastases. Optimizing fractionation choices can improve patient outcomes, reduce treatment burden, and enhance adherence to evidence-based practices. Further research is needed to develop decision-support tools for better clinical practice and patient-centered care.

## Introduction

Palliative radiotherapy (RT) has a well-established role in managing pain and symptoms for patients with advanced and metastatic cancer, with more than 50% of patients receiving radiotherapy being treated with palliative intent.^1^ Radiotherapy can effectively alleviate pain from bone metastases in up to 80% of patients, with approximately 35% experiencing complete pain relief.^2^ Additionally, palliative RT may be used to improve neurological function or prevent further deterioration in patients with brain and/or spinal cord metastases, as well as reduce symptoms caused by tumor obstruction.^1^ In Saudi Arabia, the relatively low uptake of screening programs has resulted in a higher number of cancer patients diagnosed at advanced/metastatic stages of the disease compared to Western countries.^3,4^

Single fraction palliative RT has been shown to result in equal pain relief compared to multifraction treatments for painful bone metastases^5–7^ and is recommended by consensus guidelines.^8,9^ However, studies evaluating utilization patterns in various countries have reported wide variations in practice and low uptake of single fraction radiotherapy.^10–19^ For patients with limited life expectancy, undergoing protracted treatments may impact their quality of life.

Few studies have assessed palliative RT utilization patterns in Saudi Arabia. This study aims to evaluate practice patterns among patients receiving palliative RT over six years at one of the largest institutes in the country. In particular, the study will focus on single fraction versus multifraction palliative RT and the factors associated with their utilization. Survival outcomes will also be compared between the two fractionation types.

## Materials and Methods

### Data collection

Electronic medical records at our radiotherapy department were used to identify all patients receiving palliative RT between 2018 and 2023 at King Faisal Specialist Hospital & Research Center (KFSH&RC), Jeddah. The data collected included patient’s sex, age (at the time of treatment), city of residence, primary tumor, admission status (inpatient or outpatient), date of CT simulation, target site, dose and number of fractions, radiotherapy technique (3DCRT, IMRT, or VMAT), dates of first and last fractions, whether or not patients completed their course of treatment, whether or not the patient had previous radiotherapy, and the recorded date of death. Unfortunately, patients’ performance status at treatment was not reported in patients’ charts.

### Data preprocessing

Treatment courses were categorized into single and multifraction treatments. For the purpose of this study, if multiple sites were treated at the same time, this was considered as one treatment course. Whenever both single and multifraction treatments were simultaneously used, this was considered a multifraction treatment course.

### Descriptive and survival analysis

The primary outcome was the proportion of patients receiving single versus multifraction radiotherapy. Factors potentially affecting the choice of fractionation such as patient age, sex, primary tumor, target site, year of treatment, admission type, whether or not patients had previous radiotherapy and patients’ geographical remoteness from the treatment center were evaluated. Univariate analyses using the chi-square tests were conducted to assess the effect of different factors on the likelihood of receiving single versus multifraction RT.

Survival rates were calculated based on the duration between patients’ consultation and their recorded date of death. Kaplan–Meier survival curves and log-rank tests were used to assess factors affecting patients’ survival following treatment. Additionally, we identified patients who died within 30 days after treatment and evaluated potential factors associated with this event using chi-square tests. Statistical significance, was defined as a two-tailed *p* value of <0.05. Statistical analyses were performed using IBM SPSS Statistics Software (version 29.0.2). The study was approved by the local ethics committee.

## Results

### Patient demographic and clinical data

**FIG. 1.**
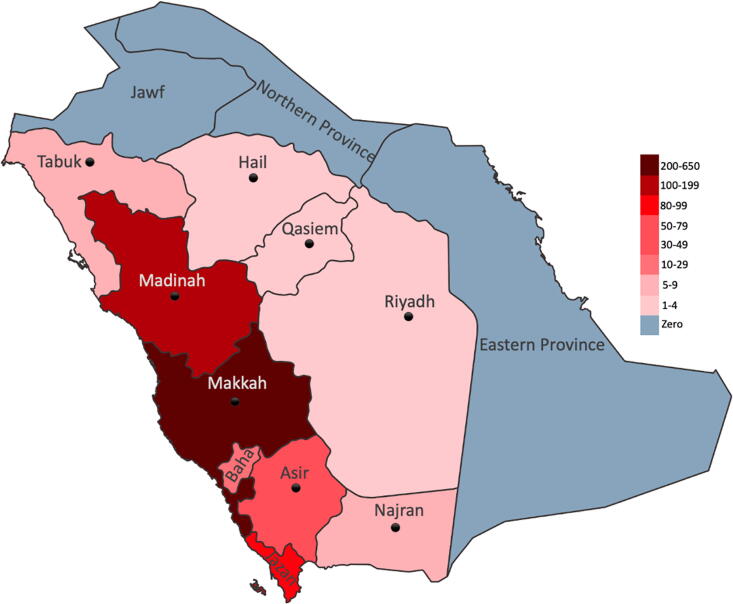
A map showing the location of the center and the number of patients from each province in the country.

A total of 792 patients received 990 palliative RT treatments between 2018 and 2023. 478 (60%) of patients were female and 314 (40%) were male. The median age (at the time of treatment) was 56.5 years (standard deviation [SD] 16.4, range 2–110). A total of 17 patients were under 18 years. Patients were similarly distributed across different age groups, with 18% under 40 years old, 18% between 40 and 49, 24% between 50 and 59, 23% between 60 and 69 and 19% were 70 years or older. The most common primary histology was breast (34%), followed by gastrointestinal (13%), genitourinary (11%), lung (11%) and head-and-neck (H&N) (10%). 11% of patients were admitted to the hospital at the time of treatment, while the remaining were treated as outpatients. Approximately half of the patients (52%) lived within 50 km from the center, while 12%, 19%, and 17% lived within 50 − 249 km, 250 − 449 km and ≥450 km from the center, respectively (Table 1). Figure 1 shows the location of the center and the number of patients coming from each province in the country.

**Table 1.** Factors Associated with Utilization of Single Versus Multifraction Radiotherapy

	Single	Multi	Univariate analysis	
	284 (28.7%)	706 (71.3%)	Or 95% CI	*p* value
Age				0.76
<40	40 (25%)	118 (75%)	1.16 (0.72–1.87)	0.55
40–49	52 (30%)	123 (70%)	0.93 (0.59–1.46)	0.75
50–59	66 (28%)	170 (72%)	1.01 (0.66–1.55)	0.96
60–69	73 (31%)	160 (69%)	0.86 (0.57–1.31)	0.49
≥70	53 (28%)	135 (72%)	Reference	Reference
Sex				0.56
Female	183 (29%)	441 (71%)	0.92 (0.69–1.22)	0.56
Male	101 (28%)	265 (72%)	Reference	Reference
Primary				0.06
CNS	9 (15%)	53 (85%)	2.36 (0.72–7.68)	0.12
Breast	111 (33%)	227 (67%)	0.82 (0.31–2.17)	0.69
Gastro	34 (27%)	91 (73%)	1.07 (0.38–2.99)	0.90
Genitalia	39 (35%)	73 (65%)	0.75 (0.27–2.08)	0.58
GYN	14 (26%)	40 (74%)	1.14 (0.37–3.52)	0.82
H&N	23 (24%)	73 (76%)	1.27 (0.44–3.65)	0.66
Lung	24 (23%)	80 (77%)	1.33 (0.47–3.81)	0.59
Other	24 (31%)	54 (69%)	0.90 (0.31–2.60)	0.85
Skin	6 (29%)	15 (71%)	Reference	Reference
Target Site				<0.001
Bone Metastases	216 (43%)	287 (57%)	1.03 (0.61–1.75)	0.91
Abdomen	10 (29%)	24 (71%)	0.57 (0.23–1.39)	0.22
Axilla	2 (17%)	10 (83%)	0.27 (0.06–1.35)	0.11
Bladder	3 (33%)	6 (67%)	0.69 (0.16–2.99)	0.61
Brain	10 (5%)	210 (95%)	0.07 (0.03–0.15)	<0.001
Breast	3 (10%)	28 (90%)	0.15 (0.04–0.53)	<0.001
Endometrium	1 (11%)	8 (89%)	0.17 (0.02–1.45)	0.11
H&N	4 (7%)	55 (93%)	0.10 (0.03–0.31)	<0.001
Lung	3 (16%)	16 (84%)	0.26 (0.07–0.97)	0.05
Prostate	1 (33%)	2 (67%)	0.69 (0.06–7.95)	0.76
Other	4 (15%)	23 (85%)	0.24 (0.07–0.77)	0.02
Multiple	27 (42%)	37 (58%)	Reference	Reference
Previous RT				0.16
No	170 (27%)	456 (73%)	1.22 (0.92–1.62)	0.16
Yes	114 (31%)	250 (69%)	Reference	Reference
Admission				0.89
Outpatient	253 (29%)	631 (71%)	0.97 (0.62–1.51)	0.89 (0.91)
Inpatient	31 (29%)	75 (71%)	Ref (Ref-Ref)	Ref
Distance (km)				0.32
<50	157 (30%)	362 (70%)	Reference	Reference
50–249	36 (31%)	79 (69%)	0.95 (0.62–1.47)	0.82
250–449	52 (28%)	137 (72%)	1.14 (0.79–1.65)	0.48
≥450	39 (23%)	128 (77%)	1.42 (0.95–2.13)	0.09
Technique			0.00 (0.00–0.00)	<0.001
3DCRT	240 (40%)	359 (60%)	Reference	Reference
IMRT	40 (13%)	279 (87%)	4.66 (3.22–6.75)	<0.001
VMAT	4 (6%)	68 (94%)	11.37 (4.09–31.57)	<0.001
Year				<0.001
2018	30 (24%)	96 (76%)	1.35 (0.82–2.23)	0.24
2019	20 (13%)	129 (87%)	2.72 (1.57–4.73)	<0.001
2020	48 (35%)	88 (65%)	0.77 (0.49–1.22)	0.27
2021	57 (34%)	109 (66%)	0.81 (0.52–1.24)	0.33
2022	64 (33%)	130 (67%)	0.86 (0.57–1.30)	0.47
2023	65 (30%)	154 (70%)	Reference	Reference
Completed RT				<0.001
Yes	274 (31%)	623 (69%)	3.65 (1.87–7.14)	<0.001
No	10 (11%)	83 (89%)	Reference	Reference

H&N, head-and-neck; RT, radiotherapy.

### Radiation treatment

**FIG. 2.**
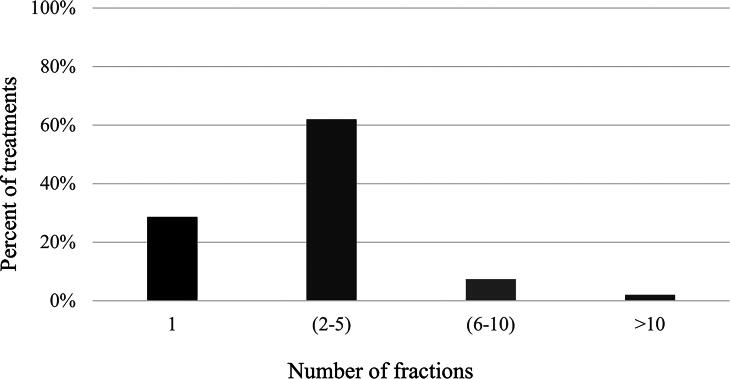
Percent of different radiotherapy fractionation regiments.

**FIG. 3.**
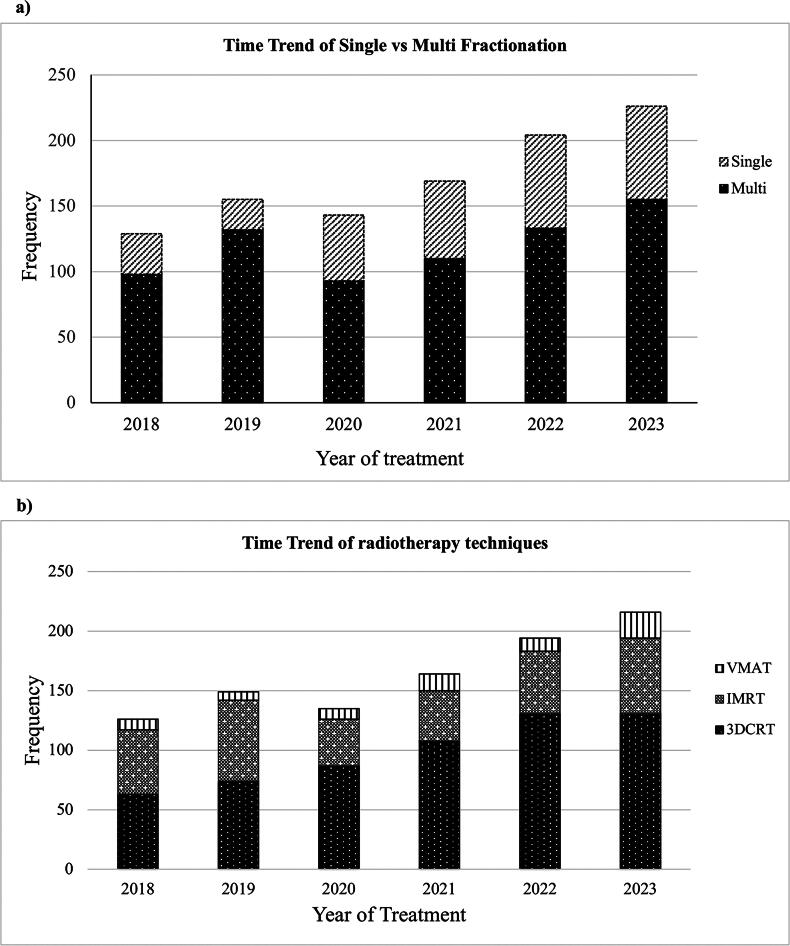
Time-trend analysis of **(a)** single fraction versus multifraction radiotherapy and **(b)** different radiotherapy techniques, over the six-year period.

Out of the 990 treatment courses, 706 (71.3%) were multifractionated and 284 (28.7%) were single fraction treatments (Table 1). Of the multifractioned treatments, 87% were 2–5 fractions, 10% were 6–10 fractions and 3% were more than 10 fractions (Fig. 2). Multifraction treatments were more commonly used for brain, breast, and H&N (*p* < 0.001). For bone metastases, 43% of cases were treated with a single fraction while 57% were treated with multifraction treatments (*p* = 0.91). Patient age, sex, primary tumor, and distance from the center did not significantly affect single versus multifraction radiotherapy utilization (Table 1). A time-trend analysis showed the overall number of patients receiving palliative RT to increase between 2018 and 2023. Still, the proportion of those receiving single versus multifraction treatments has remained between 24% and 35%, except in 2019 when it decreased to 13% (Fig. 3a).

3DCRT was the most common radiotherapy technique used in 599 (60.5%) of treatments, while IMRT and VMAT were used in 319 (32.2%) and 72 (7.3%), respectively (*p* < 0.001) (Table 1). Stereotactic body radiation therapy (SBRT) was used for only about 4% of patients. In the time-trend analysis, 3DCRT, IMRT, and VMAT proportions remained relatively constant over time with no significant change (Fig. 3b).

The most common treatment targets were bone metastases (51%) followed by brain (22%). Of the 503 bone metastases, 45% were in the spine and 28% were in the pelvis. Around 6% of patients received treatment for multiple targets simultaneously. 63% of patients received previous radiotherapy. The majority of patients (91%) completed their radiotherapy treatment (Table 1). Of the 93 patients who did not complete their treatment, 83% were prescribed multifraction treatments, while 11% were prescribed single fraction treatments (*p* < 0.001). The most cited reasons for not completing radiotherapy were poor performance status (50%), patient-related reasons (e.g., refusal or no-show) (18%), and death (17%).

The median time between the oncologist’s request and the patients’ CT simulation session was 14 days (SD 11.9, range 0–121). In 44% of treatments, the duration between the oncologist’s request and the date of simulations was 14 days, while in 10% of patients, this duration was longer than 30 days (Supplementary Appendix). There was no significant difference between single versus multifraction treatment schedules in terms of waiting time between request and CT simulation; the median waiting time was 14 days for both fractionation types. Regarding time between simulation and the treatment commencement, the median time was 7 days (SD 6.5, range 0–61). In 96% of cases, radiotherapy started in 21 days or less (Supplementary Appendix). There was a significant difference between single and multifraction radiotherapy, with the median time being 4 days for single fraction and 7 days for multifraction radiotherapy (*t* tes*t p* < 0.001).

### Survival

Only 371 patients had a recorded date of death; of those 100 received single fraction treatments and 271 received multifraction treatments. The median survival between patients’ consultation and date of death was 7.8 months (SD 9.0 months). The majority of patients (62.5%) died within 200 days (6.6 months) from date of consultation (Supplementary Appendix). Thirty-day mortality (i.e., dying within 30 days after treatment) was observed in 92 (25%) patients.

**FIG. 4.**
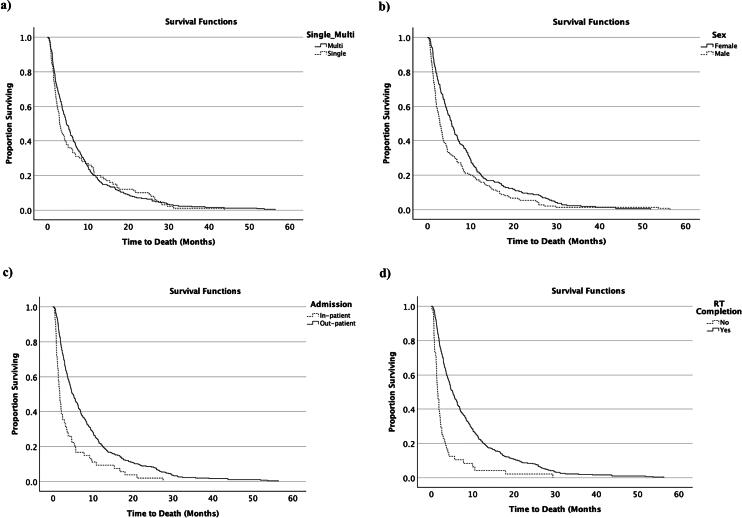
Kaplan–Meier survival (months) after radiotherapy by **(a)** Fractionation type (log-rank *p* = 0.489), **(b)** Sex (log-rank *p* = 0.002), **(c)** Admission status (log-rank *p* < 0.001), and **(d)** Whether or not patients completed their radiotherapy (RT) course of treatment (log-rank *p* < 0.001).

There was no significant difference in the median survival between patients who received single versus multifraction treatments (log-rank *p* value = 0.685) (Fig. 4a). However, median survival was shorter for males (88 days, SD 259) compared to females (169.5 days, SD 274) (log-rank *p* < 0.05) (Fig. 4b). Median survival was also shorter for inpatients (51.5 days, SD 172) compared to outpatients (151 days, SD 297) (log-rank *p* < 0.001) (Fig. 4c) and for patients who did not complete the treatment (47.5 days, SD 150) compared to those who did complete their course of treatment (157 days, SD 277) (log-rank *p* < 0.001) (Fig. 4d).

Mortality 30 days following radiotherapy was more common among males compared to females (27% vs. 23%, respectively, *p* < 0.001), among inpatients compared to outpatients (50% vs. 21%, respectively, *p* < 0.001) and among patients who did not complete their treatment compared to those who completed their treatment (63% vs. 19%, respectively, *p* < 0.001). (Table 2).

**Table 2.** Mortality Rates 30 Days After Radiotherapy

	Number of courses	Death within 30 days		
	371 total	No	%	*p* value
Fractionation				
Single	100	27	27%	0.551
Multi	271	65	24%	
Age (y) at RT				
<40	51	19	37%	0.042
40–49	63	10	16%	
50–59	86	25	29%	
60–69	97	25	26%	
≥70	74	13	18%	
Sex				
Male	220	51	23%	<0.001
Female	151	41	27%	
Primary				
Breast	109	14	13%	0.002
CNS	24	5	21%	
Gastro	64	25	39%	
Genitalia	38	11	29%	
GYN	30	3	10%	
H&N	30	10	33%	
Lung	45	14	31%	
Skin	4	0	0%	
Other	27	10	37%	
Target Site				
Bone metastases	181	57	31%	0.031
Abdomen	14	4	29%	
Axilla	6	1	17%	
Brain	82	14	17%	
Endometrium	6	1	17%	
H&N	23	6	26%	
Lung	6	1	17%	
Other (rectum, cervix, bladder, breast)	25	1	4%	
Multiple	28	7	25%	
Previous RT				
Yes	134	28	21%	0.191
No	237	64	27%	
Admission				
Inpatient	54	27	50%	<0.001
Outpatient	317	65	21%	
Distance (km)				
<50	233	57	24%	0.937
50–249	35	8	23%	
250–449	61	17	28%	
≥450	42	10	24%	
Technique				
3DCRT	219	60	27%	0.282
IMRT	131	29	22%	
VMAT	21	3	14%	
Year				
2018	46	16	35%	0.045
2019	60	13	22%	
2020	58	9	16%	
2021	63	15	24%	
2022	74	14	19%	
2023	70	25	36%	
Completed RT				
Yes	323	62	19%	<0.001
No	48	30	63%	

H&N, head-and-neck; RT, radiotherapy.

## Discussion

Practice patterns of single and multifraction palliative RT were reviewed at one of the largest tertiary hospitals in the country. To our knowledge, this has yet to be assessed in our population. Reviewing practice patterns is crucial for ensuring adherence to clinical guidelines, identifying areas of improvement, and improving patient care.

Of all radiotherapy courses delivered between 2018 and 2023, approximately 29% were single fraction, while 71% were multifraction treatments with little change over time. Looking at only cases of bone metastases, 43% were treated with a single fraction while 57% were treated with multifractionation RT. These findings are consistent with other reports from other countries.^20,21^ Higher rates of single fraction radiotherapy utilization for bone metastases (44%–65%) were reported in the United Kingdom^22^ and Canada^18,23,24^ while slightly lower rates (30%) were reported in Australia.^10,16^ Much lower rates (3%–8%) were observed in the United States, with more than 50% receiving 10 or more fractions.^11,25–27^ This is despite practice guidelines recommending single fraction radiotherapy, particularly for patients with poor prognosis and those with transportation difficulties.^28^

Of the multifraction treatments, most treatments in our center (87%) were delivered in 2–5 fractions while 10% and 3% were delivered in 6–10 and over 10 fractions, respectively. This followed the ASTRO Choosing Wisely guideline recommendation of limiting the number of patients receiving 10 fractions or more.^28^

Patients receiving radiotherapy to extremities were more likely to receive single fraction treatment compared to other target areas (*p* < 0.05), while those treated for H&N and brain tumors were more likely to receive multifraction treatments (*p* < 0.001). A possible explanation for this could be the increased risk of toxicities when treating the brain and H&N regions compared to the risk when treating extremities. While use of single fraction treatments for bone metastases is well-supported by evidence, its role in nonbony sites—particularly H&N—requires further investigation. Alternative hypofractionated regimens, such as quad shot, may offer a balance between symptom relief and treatment burden for these cases.^29,30^ No other factors were significantly associated with single fraction. Other studies have observed higher utilization rates of single fraction radiotherapy for patients over 80 years old^10,17,31^ and those living in remote areas.^23^ However, such findings were not observed in our cohort.

Distance to the treatment center did not affect clinicians’ choice of fractionation even though 36% of treatments were received by patients who lived 250 km away. The use of multifraction radiotherapy for such patients could be due to them choosing to stay locally during the course of their treatment and clinicians’ concern about the potential need for re-irradiation. However, this could not be ascertained without further investigation. Patients from rural areas can greatly benefit from single fraction treatments in terms of logistics. Rapid response radiotherapy clinics where patients have their simulation and treatment in one day have been shown to increase radiotherapy utilization and the choice of single fraction treatments in this patient cohort.^32,33^

It has been suggested that private health insurance reimbursement policies could influence clinicians’ choice of multifraction over single fraction treatments.^34,35^ This, however, does not apply to our cohort, as the government fully subsidized all treatments. Therefore, clinicians’ choice between single or multifraction radiotherapy was not affected by the cost of treatment.

Another potential factor for choosing fractionation types could be the pressure of long waiting lists. Clinicians working under the pressure of limited time and long waiting lists may prescribe single fraction rather than multifraction treatments in cases where both are appropriate. However, an analysis of waiting times between referral and patient start dates did not confirm this variation. Similar findings were reported by Haddad et al. (2005).^19^

Regarding patients’ survival, no significant difference was found between patients receiving single fraction and those receiving multifraction radiotherapy. However, significantly shorter median survival was observed for males compared to females (88 days vs. 169.5 days, *p* = 0.002). This could be due to the high proportion of breast cancer in our sample resulting in higher survival rates for females. Shorter median survival was also observed in patients admitted to the hospital during their treatment compared to those treated as outpatients (1.7 months vs. 5 months, *p* < 0.001). Similar results were reported by Ellsworth et al. (2014)^11^ and this could be related to other factors, including performance status and other co-morbidities. These results further suggest that single fraction treatment should be strongly considered for inpatients. Moreover, shorter median survival was observed in patients who did not complete their course of treatment compared to those who did (47.5 days vs. 157 days, *p* < 0.001), which may also be linked to other factors such as performance status and co-morbidities. Of note, the majority of patients (83%) not completing their treatment were prescribed multifraction treatments, suggesting that single fraction treatment may increase the likelihood of patients completing their radiotherapy.

Approximately a quarter of patients died within 30 days after completing radiotherapy. Of those, similar proportions received single versus multifraction treatments (29% vs. 71%), indicating that clinicians did not consider single fraction treatments for patients with limited prognosis. These findings are similar to those reported from the US^11,36^ but not from Canada^23^ and Australia^10,16^ where higher rates of single fraction radiotherapy were used among patients who died within 30 days. Patients with poor prognosis could benefit the most from single fraction treatment, as they frequently experience multiple areas of pain and mobility issues, making repeated visits to the radiotherapy center challenging and burdensome.^37^ For advanced cancer patients with painful bone metastases, an early and systematic integration of palliative care may improve symptom management, quality of life and ensure the provision of more patient-centered care.^38^

SBRT has been shown to result in outcomes equivalent to those of multifraction radiotherapy.^39^ However, it is limited to patients with good prognosis, and patients receiving SBRT should be selected appropriately.^40^ Similar to reports by Ellsworth et al. (2014)^11^ only about 4% of our patients received SBRT. Personalizing care for patients who benefit from SBRT is essential to improve cost-effectiveness.

Future directions include investigating outcomes for single fraction treatments in various clinical settings and predictors of survival after radiotherapy to help guide clinical decision-making. Using clinical pathway decision support tools has increased the utilization of single fraction radiotherapy from 18% to 48% in patients treated for bone metastases.^41^

Limitations of this study include the retrospective design and being a review of a single, large institution where the patient population may be skewed to advanced and/or refractory disease. Additionally, the lack of data on patients’ performance and comorbidity may have provided better insights into our findings, especially as other studies have reported their association with higher utilization of single fraction radiotherapy. Nevertheless, our findings captured the overall practice patterns within the department.

In conclusion, single fraction radiotherapy remains underutilized for bone metastases and should be considered more strongly in eligible patients, particularly those with limited prognosis. However, for nonbony sites, alternative hypofractionated regimens may also align with the literature and patient needs. Almost 60% of patients with bone metastases received multifraction treatments resulting in an unnecessary burden on patients, reducing their likelihood of completing therapy courses and increasing the workload on radiotherapy departments. Survival was shorter for patients admitted to the hospital at the time of treatment; therefore, shorter treatment courses should be considered in such cases. Further research is needed to increase clinicians’ ability to predict patient survival outcomes after palliative RT and to optimize single fraction schedules utilization where appropriate.

## Abbreviations Used

CNS: Central nervous system
CT: Computed tomography
3DCRT: 3-D Conformal radiotherapy
GASTRO: Gastrointestinal
GYN: Gynecology
H&N: Head and neck
IMRT: Intensity-Modulated Radiation Therapy
RT: Radiotherapy
SBRT: Stereotactic body radiation therapy
VMAT: Volumetric modulated arc therapy
