# Supplementary material for: Practice Patterns of Palliative Radiotherapy for Advanced Cancer at a Large Institute in Saudi Arabia
Source: Palliat Med Rep. 2025 Apr 29;6(1):223–32. doi: 10.1089/pmr.2025.0008 (PMC12411897; doi:10.1089/pmr.2025.0008)
Supplement: Supplementary Appendix [file pmr.2025.0008_supplementaryappendix.docx]

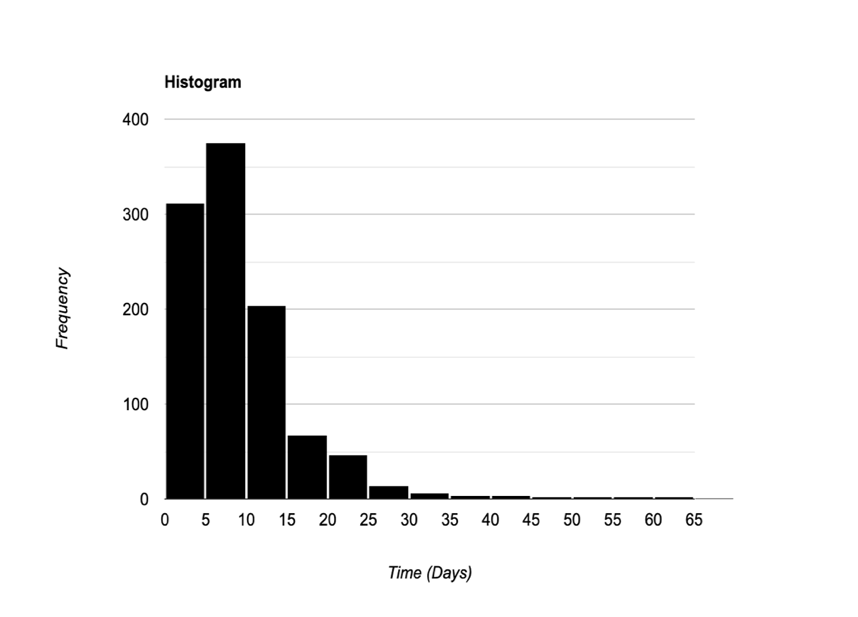

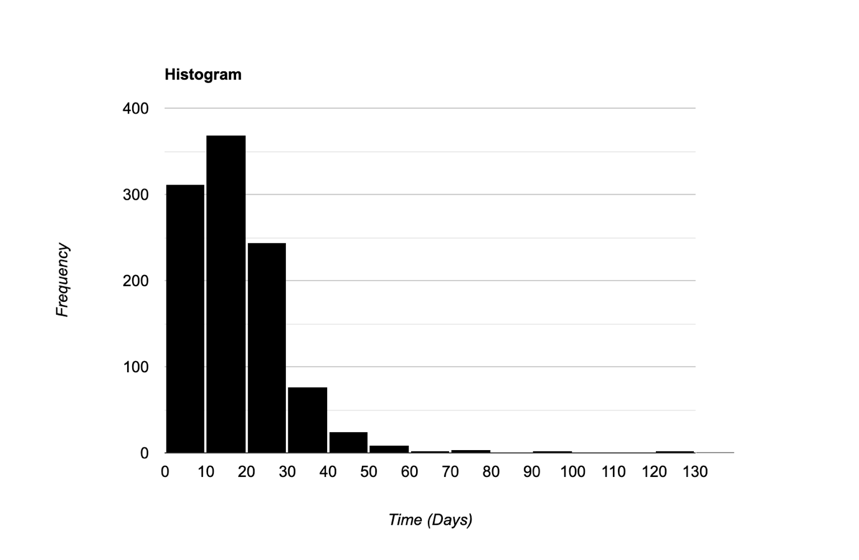


**a) b)**

**Appendix:1** Histogram of the time between a) clinician request to the date of the CT simulation and b) CT simulation to the date of the first fraction.


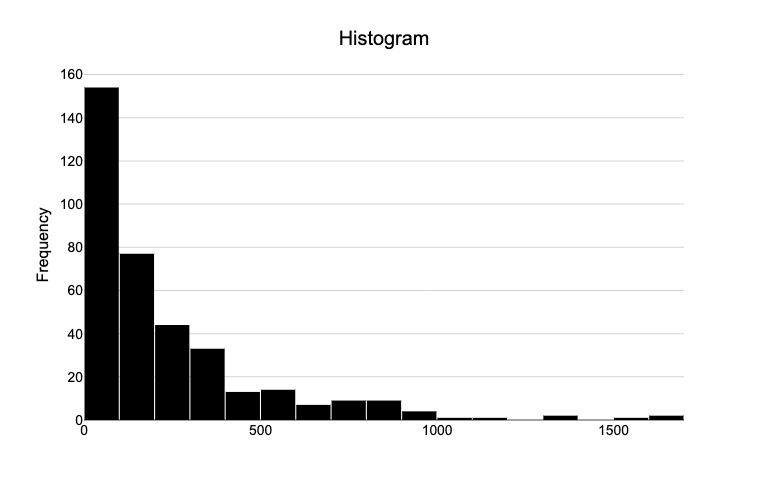


*Time (Days)*

**Appendix: 2** Histogram of the time between date of consultation and date of death.
